# Honest advocacy for nature: presenting a persuasive narrative for conservation

**DOI:** 10.1007/s10531-016-1163-1

**Published:** 2016-06-30

**Authors:** David C. Rose, Peter N. M. Brotherton, Susan Owens, Thomas Pryke

**Affiliations:** 1 Present Address: Department of Geography, University of Cambridge, Downing Place, Cambridge, CB2 3EN UK; 2 Natural England, Unex House, Peterborough, PE1 1NG UK

**Keywords:** Evidence-based policy, Evidence-informed policy, Framing, Science communication, Science-policy interface

## Abstract

Conservation scientists are increasingly recognising the value of communicating policy-relevant knowledge to policy-makers. Whilst considerable progress has been made in offering practical advice for scientists seeking to engage more closely with decision-makers, researchers have provided few tangible examples to learn from. This paper uses an English case study, but draws out important high-level messages relevant to conservation scientists worldwide. The case study looks at how the *Lawton Review* presented knowledge persuasively about the suitability of England’s ecological network to deal with future pressures. Through skilful framing of rigorous scientific knowledge it was able to make a significant impact on government policy. Impact was achieved through: (1) selecting politically salient frames through which to communicate; (2) using clear, accessible language, and; (3) conducting rigorous science using an authoritative team of experts. Although its publication coincided with a favourable policy window, the *Lawton Review* seized on this opportunity to communicate a rigorously argued, persuasive and practical conservation message; in other words, it performed ‘honest advocacy’. Thus, whilst it remains important to conduct scientific research with technical rigour, conservation scientists could also benefit from identifying salient frames for conservation and communicating clearly.

## Introduction

Policy analysts have long recognised that the framing of knowledge is a significant factor in determining the outcome of science-policy interactions (Hajer 2003; Owens 2015; Rein and Schön 1991; Rose 2015). By framing knowledge astutely, it can enhance the policy relevance of research, particularly if proposed solutions can be made to fit within existing political priorities. Clear communication is also an important part of engaging well with policy-makers who are not expert in the field (Torgerson 1986). As part of efforts to engage more closely with policy, conservation scientists have also begun to identify the usefulness of frames, or narratives, through which to enhance the influence of their knowledge (Carmen et al. in prep.; Cook et al. 2013; Howard et al. in prep.; Jokinen et al. in prep..; Leslie et al. 2013; Lawton and Rudd 2014; Rose 2015; Tinch et al. in prep.). Indeed, there has been a marked increase in inter-disciplinary collaborations between conservation scientists and policy researchers (Jørgensen et al. 2014; Sarkki et al. 2014, 2015; Young et al. 2014), exemplifying the progress made since Agrawal and Ostrom’s (2006) claim that conservation was ambivalent towards political science.

Furthermore, conservation scientists have sought to offer useful advice about how to engage better with policy (Burgman 2015); ranging from the early identification of forthcoming issues on the policy agenda to prepare relevant research in advance (Sutherland et al. 2015a) through to more fundamental issues concerning the interdisciplinary training of graduate students (Blickley et al. 2013; Bainbridge 2014). In light of such recommendations, it is clear that some conservation researchers are starting the necessary process of identifying practical methods to overcome the limited uptake of knowledge into policy. However, despite these useful contributions, little research in conservation science has focused on a specific case where skilfully framed knowledge has been influential in decision-making; indeed, a review by Spruijt et al. (2014) suggests that there is a lack of empirical evidence for interactions at the science-policy interface across all disciplines.

This paper provides an empirical example of knowledge impact, and explores how it might help conservation scientists to frame and communicate research effectively. Whilst it uses an English case study, it seeks to draw out lessons for conservation scientists working elsewhere. Specifically, it focuses on the impact of one report published in 2010, which had an immediate effect on government policy in England, partially as a result of an astute framing of knowledge. In fact, this report had such a profound influence on policy that it is widely seen as ‘an example of how to present good science to policy-makers’ (*Department for Environment, Food and Rural Affairs [DEFRA] civil servant 2, in interview for this project*). In examining the case, this paper builds on science-policy work that stresses the need for practical advice, but takes the debate one step further. Instead of merely identifying methods through which conservation scientists could achieve greater engagement with policy, it provides a solid example for researchers to grasp, interrogate, and take lessons from. It does not suggest that the events described can be replicated, nor can the *Lawton Review* be seen as a ‘logical framework’ (*in* sensu Black and Copsey 2014) from which scientists should not diverge. Instead, this paper stresses the importance of framing and communication to improve the salience of scientific evidence. In doing so, it shows that it is possible to be an honest advocate for nature by presenting rigorous science in a convincing way.

## The *Lawton Review—*achieving a ‘direct hit’

The report ‘Making Space for Nature: A Review of England’s Wildlife Sites and Ecological Network’ (hereafter referred to as the *Lawton Review*) was produced by an independent Review panel in 2010, and assessed the coherence of England’s ecological network and its capability to withstand future pressures (such as those linked to climatic and land use changes). The *Lawton Review* promoted the idea of landscape-scale conservation, and summarised its 24 recommendations by the four-word phrase *‘More, Bigger, Better, Joined’*. It presented a strong scientific case for a wider approach to conservation in England, moving away from a dominant site-based strategy focused on individual reserves towards a more holistic concept.^1^

The *Lawton Review* was commissioned in the final year of a Labour-led government and was ultimately submitted to a Conservative-led coalition government. Despite the change in political administration, the *Lawton Review* achieved an immediate and significant impact on government policy affecting England, representing a key part of the knowledge underpinning the *Natural Environment White* Paper^2^ (DEFRA 2011a; Lawton and Rudd 2016). In the UK, a White Paper reflects a government’s intention to tackle particular problems by preparing policies which may be enacted into legislation (Shin and Choi 2014). This was the first White Paper to deal specifically with the natural environment and conservation for over twenty years. The *Natural Environment White Paper* (and the related *Biodiversity 2020* strategy, Defra 2011c), published less than a year after the *Lawton Review,* endorsed a landscape-scale approach as the government’s policy position. The statements below illustrate the impact of the *Lawton Review* on the subsequent White Paper:

The [Lawton] report was one of the key drivers of our *Natural Environment White Paper* published in June 2011, and the England Biodiversity Strategy published later in 2011. (Nick Clegg, Deputy Prime Minister 2011 cited in Harper 2012).

The Lawton Report, Making Space for Nature, found that nature in England is highly fragmented and unable to respond effectively to new pressures such as climate…Past action has often taken place on too small a scale. We want to promote an ambitious, integrated approach, creating a resilient ecological network. (*Natural Environment White Paper*, DEFRA 2011a, p. 5)

Although making an impact on government and institutional policy does not guarantee a net conservation benefit on the ground, it is a first step to achieving an impact in practice.

Thus, the *Lawton Review* seemed to provide a compelling scientific base for the landscape-scale strategy set out in the White Paper (Adams et al. 2014). Its rapid and direct policy impact stands in contrast to that of many other scientific advisory reports. For example, a report with similar scope to the *Lawton Review* published ten years previously (Hossell et al. 2000) also recommended the move towards a landscape-scale approach, but seemed to have little direct impact on government policy (*based on an interview of one of its co*-*authors for this project*). The failure to have immediate impact is not in itself surprising; such an outcome has often been observed by analysts of policy advice. For example, Owens’ (2015) study of the UK Royal Commission on Environmental Pollution (which advised governments over 40 years) showed that whilst some reports had immediate effect (and a few never gained traction), influence was often a diffuse affair involving cognitive and discursive processes over extended periods of time.

The significance of the immediate impact experienced by the *Lawton Review* is strengthened because the need for landscape-scale conservation had been consistently communicated to policy-makers for at least twenty years (Adams et al. 2014). It is therefore interesting to ask why the *Lawton Review* experienced an immediate and significant impact on government policy. Illuminating the reasons for this impact may help conservation scientists to improve the policy impact of their work, ultimately improving the chances for evidence-based conservation.

## Methods

### Documentary analysis

A thematic analysis was conducted on the *Lawton Review* (Lawton et al. 2010) and related to two key areas. Firstly, it asked how the review framed reasons to conserve biodiversity. Secondly, it asked at what governance levels a landscape-scale approach to conservation should be carried out and whether examples of success stores were used. These two areas were selected on the basis that persuasive narratives for the conservation of biodiversity are important for policy change, as are suggested policy mechanisms that are practical to carry out. Two broad motivations for conserving nature were found after coding the document^3^—(1) nature provides valuable ecosystem services and (2) nature has intrinsic value. On the second question, the broad thrust of the *Lawton Review* emphasised the need for local, and multi-stakeholder, contributions to landscape-scale conservation and used success stories to illustrate where landscape-scale conservation had worked.^4^ Keywords were developed for these five themes (see Table 2)—ecosystem services, intrinsic value of nature, localism, diverse stakeholders, and success stories, as well an additional theme of climate change (found to be emphasised throughout from an initial read-through)—and were counted to see how many times they appeared in the review (a similar ‘word use’ process to Admiraal et al. 2016). The same keywords were then counted to find how many times they appeared in the *Natural Environment White Paper* (DEFRA 2011a) and its associated evidence report (DEFRA 2011b) was counted. This process, shown in Table 2, was undertaken to investigate the saliency of key themes in the *Lawton* *Review* as compared to the subsequent White Paper.

### Semi-structured interviews

Semi-structured interviews were carried out as part of a wider PhD project that assessed the impact of landscape-scale conservation as an idea on UK policy from 1990 to 2011. Thirty-five interviewees (Appendix 1) were chosen based on their ability to discuss science-policy interactions at all levels of governance: national government/statutory agency level (UK and England), local government, as well as others working at the conservation-science policy interface, including members of conservation NGOs and academics. Interviews lasted up to an hour and were tailored to some extent to the individual.

The first part of the interview (see Appendix 2) covered general questions about the relationship between science and policy, focusing on barriers to evidence uptake and the role of frames and storylines in evidence communication. The latter part of the interview focused on the *Natural Environment White Paper*, asking about the reasons for the endorsement of landscape-scale conservation and discussing the reasons for the immediate impact of the *Lawton Review*. All interviewees were able to contribute fully on the section devoted to general barriers to evidence uptake, but four interviewees with more practice-based mandates were generally less able to comment on the fortunes of the *Lawton Review*. The motivations for the White Paper content and the impact of the *Lawton Review* were best discussed with participants who were closely associated with both documents. These included DEFRA Ministers/civil servants, Natural England staff, Professor Sir John Lawton, and to some extent academics who had knowledge of the policy events. Other senior members of conservation organisations had also followed the progress of the *Lawton Review* and the White Paper and were able to give informed comment about the reasons for impact. Quotations used for the impact of the *Lawton Review* on the White Paper, therefore, tend to be selected from those who had the greatest knowledge of events. Each interview was recorded, transcribed in full, and coded manually to look for key themes in the data.^5^ As this paper is focused on the *Lawton Review*, the codes produced from an analysis of the second part of the interview are included here (Table 1).

**Table 1:** Coding document from analysis of interviews

Initial codes	Merged codes	Final codes
Ecosystem services	Ecosystem services politically salient	Ecosystem services politically salient
Economy trumps everything, particularly in austerity		
Timing of evidence	Political context matters for evidence uptake	Political windows for evidence uptake
Policy windows		
Political will		
People in power matter		
Implementation of White Paper poor	Poor implementation of White Paper	*DROPPED* *(Mentioned less often*—*not focus of this paper)*
Good communication	Framing and use of language important	Framing and use of language important
Framing of evidence important		
Observed changes important	Success stories	Success stories
Practice influences policy		
Bottom-up communication		
Climate change affects nature	Climate change salient and important for conservation	Climate Change salient political issue
Climate change emergent in conservation		
Climate change is salient political issue		
Localism/civil society role	Localism	Localism
Communication of uncertainty	Good science, certainty, authority	Level of and framing of certainty
Quality and authority of science		
Power of good quantitative science		

## Results

Overall, interviewees considered several factors to be important in explaining the impact of the *Lawton Review* (Table 1)—including the economic valuation of nature (and other services), a favourable policy window, good framing and use of language, the presentation of success stories, the use of emblematic climate change, localism, and the rigour and certainty of the science.

Some of these factors mirror the identification of common themes used in both the *Lawton Review* and the *Natural Environment White Paper* (Table 2); which were the economic value of nature (and other services), climate change, localism, and the inclusion of stakeholders.

**Table 2:** Documentary analysis results

Themes	Keywords	Number of times keywords mentioned in Lawton review	Number of times keywords mentioned in White Paper	Number of times keywords mentioned in White Paper evidence report
Ecosystem services/natural capital	Ecosystem services/economy/natural capital	64	242	220
Intrinsic value of nature	Intrinsic	3	2	2
Localism	local authorit/local government/local communit	54	71	6
Success stories/good news	Success/good news	19 (inc. 6 boxes)	7	2
Including stakeholders	Stakeholder[s]/business[es]/ conservation organisation[s]/landowner[s]/ farmer[s]/citizen[s]	93	195 (dominated by business)	102 (dominated by business)
Climate Change	Climate change	76 (and 2 special sections)	59	16

These findings can be neatly discussed in four sections to determine why the *Lawton Review* was influential–(1) the use of politically salient frames to illustrate the value and practicality of landscape-scale conservation, (2) the use of clear, accessible language, (3) the power of good, rigorous science conducted by an authoritative group of experts, and (4) the juxtaposition of the review’s publication with a favourable policy window.

Upon tracing the previous academic contribution of Professor Sir John Lawton to policy analysis, it is suggested that the use of salient frames, clear accessible language, good science, and the ability to seize on a policy window were deliberate techniques. Notably, Lawton reported that his team received no interference from policy-makers in terms of how to write the review. In an interview for this study, Lawton argued:

Nobody told us how to write the review. I took the very conscious decision, with the full support of my panel, to write it in plain English, with ‘good news stories’ where they were appropriate, and a persuasive, interesting narrative that was scientifically accurate.

In an earlier paper, Lawton (2007) attempted to answer questions commonly faced by conservation scientists. He wrote:

My ultimate aim is simple: to make sure that when ecologists do enter the political arena they do so with their eyes open, expecting to be in it for the long haul in a process that is messy, complex and iterative. (Lawton 2007, p. 465)

As chair of the Royal Commission on Environmental Pollution (RCEP) (2005–2011), Lawton was able to gain considerable knowledge of the policy-making process. Owens (2015) found that reports of the RCEP had widely varying effects, and thus Lawton would have been acutely aware of the complex and contingent relationship between science and policy. Hence, he was in a position to act as a key policy entrepreneur by making use of his knowledge of the science-policy interface (Lawton and Rudd 2016).

### Politically salient choice of themes in *Lawton Review*

From documentary analysis and interviewing, it is clear that the *Lawton Review* used salient frames through which to show the importance of landscape-scale conservation (Table 2).

#### Frame 1: Emblematic climate change

An academic conservation scientist noted that ‘the Lawton Report could have been written without climate change’ (Academic Conservation Scientist A), because several drivers of habitat fragmentation, such as urban sprawl, are also responsible for creating an isolated network of Protected Areas. Although the *Lawton Review* did address a number of drivers of biodiversity loss (e.g. poor habitat management, introduced pests), it chose to draw substantially on the threat of climate change to promote landscape-scale management (see Table 2). The focus on climate change is clear throughout; the quality of the ecological network is always discussed in the context of ‘climate change and other pressures’ (Lawton et al. 2010, p. 4). In fact, it argues that ‘climate change, particularly in the longer term, may have the biggest impact of all.’ (Lawton et al. 2010, p. vi). A leading scientist at Natural England also noticed this emphasis:

Of course climate change is not the only driver in the *Lawton Review*, but it certainly was an important factor in the context of the report. The *Lawton Review*’s use of climate change chimed with the landscape-scale approach to conservation.

The relative resonance of climate change with policy-makers, as compared to biodiversity conservation, has been discussed in the literature. Jørgensen et al. (2014, p. 2) suggested that climate change is the most important contemporary problem for public policy, with many considering the issue to be ‘*the* environmental issue of the twenty-first century’. This statement draws parallels with the work of Hajer (1995) who argued that specific issues can achieve emblematic status for a particular cause. From a UK perspective, climate change is arguably the most discussed environmental issue by central government, particularly after the UK Climate Change Act (2008) was passed with cross-party support. Indeed, it was hailed as the ‘greatest threat to our common future’ in the Queen’s Speech of 2010 (in Shin and Choi 2014, p. 11). In addition, Zaccai and Adams (2012) have investigated how the prominence of climate change affects its influence as a policy issue. Comparing it to the concept of biodiversity loss, they argued that climate change is ‘better defined and better understood’ (557) by policy-makers. In contrast, biodiversity loss is ‘less easily understood, less tangible, and policy responses do not engage major economic sectors’ (557).

Documentary analysis of the *Lawton Review* and White Paper further illustrated the growing political salience of climate change (Table 2). As seen in the brief given to the *Lawton Review* team:

…with the effects of climate change and other pressures on our land, now is the time to see how we can enhance ecological England further to make ecological corridors and a connected network. (Benn in Lawton et al. 2010, p. ii)

The *Natural Environment White Paper* referred to the issue widely, as did Caroline Spelman, who was in receipt of the *Lawton Review* as Secretary of State at the Department of Environment, Food and Rural Affairs (DEFRA). When asked why the *Lawton Review* had an immediate impact on policy, Spelman argued that ‘there was a greater acceptance and a greater understanding of the threats of climate change and the urgency of the problem’. Furthermore, the emphasis on climate change within the *Natural Environment White Paper* (2011) was clear:

Climate change is one of the biggest environmental threats facing the world today, and perhaps the greatest economic challenge…Helping the natural environment to adapt to climate change is a theme that runs throughout this White Paper. (*Natural Environment White Paper*, DEFRA 2011a, p. 10)

Thus, it is possible to see the relevance of climate change when the *Lawton Review* was published. The *Lawton Review* did not have to focus on climate change to make the case for landscape-scale conservation, but made a deliberate choice to do so, and hence seized on the opportunity to convey a politically salient idea.

#### Frame 2: The political salience of an economic valuation of nature

The *Lawton Review* employed a second framing of knowledge about landscape-scale conservation, arguing that its recommendations would contribute to the protection of valuable ecosystem services (Table 2). The following extract from the review illustrate this storyline:

The report argues that we need a step-change in our approach to wildlife conservation…to one of large-scale habitat restoration and recreation, under-pinned by the re-establishment of ecological processes and ecosystem services. (Lawton et al. 2010, p. .ii)

The extract highlights the strong link between the report’s landscape-scale recommendations and ecosystem services, using an economic storyline to ‘underpin’ habitat restoration. As with the incisive use of climate change as a policy-relevant narrative, the use of ecosystem services further represented a useful context. Whilst there is disagreement about the suitability of an ‘ecosystem services’ mind-set to prevent biodiversity loss (McCauley 2006; Mace et al. 2012; Adams 2014), framing conservation in such a manner is arguably necessary if voters (and governments) continue to prioritise economics (EU Commission 2013). In fact, many authors have argued that employing an ecosystem services storyline can be useful for placing conservation onto a political agenda (Daily and Matson 2008; Jørgensen et al. 2014; Tinch et al. in prep.).

This literature fits well with the thrust of government policy documents around the time of the *Lawton Review*. In fact, the focus on the economic value of nature (and other services) was the most prominent theme in the White Paper and its associated evidence report (Table 2). In a brief discussion of the *Natural Environment White Paper*, Adams et al. (2014, p. 574) outline its language of ‘growth, prosperity, security, and benefits’. They also highlight how conservation was being increasingly presented at this time as a way of achieving wider social and economic benefits (Adams et al. 2014). The knowledge report for the White Paper (DEFRA 2011b, p. 3) also stated that ‘nature represents a stock of assets from which society benefits in numerous and hidden ways’. This in turn affected the focus of the White Paper itself. It argued that ‘a healthy, properly functioning natural environment is the foundation of sustained economic growth…the reasons for many of the actions proposed in this White Paper’ (*Natural Environment White Paper*, DEFRA, 2011a, pp. 3–4).

Interviewees from DEFRA also referred to the quasi-economic content in the *Natural Environment White Paper*. Caroline Spelman, Secretary of State at DEFRA, argued that ‘the National Ecosystem Assessment gives real ability to calculate the worth of nature that we previously thought was provided for free.’ Furthermore, Professor Sir John Lawton thought that ‘quite a lot of politicians’ had begun ‘to get the fact that the environment provides services’. Lawton also reported that his team had worked closely with academics working on the UK National Ecosystem Assessment (UK NEA 2011), published around the same time. This assessment of the value of ecosystem services in the UK concluded that nature provided multi-billion pound benefits to society (UK NEA 2011), and it played an important role in increasing the political salience of biodiversity conservation.

The *Lawton Review* also framed the economic cost of doing landscape-scale conservation in a positive way. Whilst recognising that there would be a management cost, the consequences of taking early action were framed as beneficial. It argued that ‘there is one thing of which we can be certain: the sooner we act to establish a coherent and resilient ecological network, the lower the eventual cost and the greater the benefits for us all.’ (Lawton et al. 2010, p. ix). Alongside the wider framing of ecosystem services, this helped knowledge about landscape-scale conservation to be received in a positive light by policy-makers.

#### Frame 3: Localism

A further storyline used in the *Lawton Review* referred to a localist approach to governance, in which locally-led decisions (not central government dominated) were needed for effective landscape-scale management. The following extract from the report illustrates the widespread promotion of local approaches to conservation:

Delivering our vision is not a job for government alone…We will not achieve a step-change in nature conservation in England without society accepting that it is necessary, desirable, and achievable…Many of the decisions on the priorities for action are best made locally. (Lawton et al. 2010, p. 3)

With reference to the *Lawton Review*, framing landscape-scale conservation in terms of localism represented a further policy-relevant storyline, improving the prospects for influence. Localism was a politically mainstream issue at the time of publication, the Conservatives having fought their successful 2010 General Election in part on a localist agenda (The Guardian 2010). If, as Kingdon (2003) suggests, ideas are most influential when they can be used as ready-made policies to suit pre-existing ideologies, it seems likely that the new government were actively searching for projects through which to realise a localist agenda.

In the context of the *Localism Act* (2011), which cemented these ambitions, it is possible to witness the clear emphasis on local decision-making in the *Natural Environment White Paper* (2011). The White Paper set out to achieve landscape-scale conservation through ‘joined-up action at local and national levels to create an ecological network which is resilient to changing pressures’ (DEFRA 2011a, p. 14). It referenced the concept of localism regularly, and set up Local Nature Partnerships (LNPs):

In developing this White Paper, we have received one particularly clear message: effective action to benefit nature, people and the economy locally happens when the right people come together in partnership… We will encourage and support Local Nature Partnerships where local areas wish to establish them. (DEFRA 2011a, p. 19)

Furthermore, the shift of emphasis away from centralised decision-making was noticeable in an interview conducted with a DEFRA civil servant (3). This interviewee referred to the aims of LNPs:

‘I think we are all hopeful…for any mechanism, which seeks to inspire local delivery, that LNPs may drive policy from bottom-up…We hope that any useful, innovative measures that LNPs may identify, which DEFRA can see working, can feed back into policy development.’ (DEFRA Civil Servant 3)

Therefore, it was again pertinent to seize on a salient political issue by employing a localist storyline in the *Lawton Review*. The inclusion of non-governmental stakeholders also links here, but is discussed in the following section.

#### Frame 4: Success stories, deliverable recommendations, and the inclusion of stakeholders

The *Lawton Review* presented knowledge for landscape-scale conservation in the context of action-based success stories. It indicated that projects had already been implemented on the ground by voluntary conservation organisations, with considerable success:

‘There are…well thought through frameworks to inform and, where necessary, co-ordinate such actions [landscape-scale], including The Wildlife Trusts’ vision of a Living Landscape, the RSPB’s Futurescapes, emerging proposals for landscape-scale initiatives from the England Biodiversity Group, Regional Opportunity Maps and the Wetland Vision for England’ (Lawton et al. 2010, p. 62)

Further references were made to the lessons learned from trialling conservation projects in practice through the use of six special boxes in the review. This action-based knowledge was useful in convincing policy-makers that landscape-scale conservation would work, particularly as a practical template for landscape-scale management had been historically lacking (Morecroft et al. 2012). Lessons learned from NGO-led landscape projects during the late 2000s therefore reduced the risk of enacting a potentially risky and costly policy. A civil servant (1) in DEFRA made a similar point on the value of showing that landscape-scale conservation projects worked:

Responses from colleagues closely involved in the *Natural Environment White Paper*…suggest that they saw these kinds of projects as very much proof of concept, demonstrating that you could link up large areas of landscape…and that it was feasible…This gave the *Natural Environment White Paper* team confidence in advocating the landscape scale approach. (DEFRA civil servant 1)

This civil servant made a clear link between tested projects on the ground and the policy commitments made in the White Paper. This process mirrors much of the recent conservation literature, which indicates that positive framing can improve the policy impact of scientific knowledge (Balmford 2012; Carmen et al. in prep.), particularly if the action-based knowledge clearly illustrates ‘what works’ (Sutherland et al. 2015b). Indeed, a DEFRA civil servant (2) thought that individual policy-makers are affected by a range of knowledge types; some by graphs and equations, but others ‘need a different form of presentation, much more action-oriented, something that has been demonstrated as working’. The inclusion of action-based knowledge thus enhanced the recommendations of the *Lawton Review*.

Demonstrating that the *Lawton Review’s* recommendations were feasible was particularly important because the final price tag attached to achieving the end-goal of a ‘coherent and resilient ecological network’ was politically daunting: £600 m–£1.1 billion (Lawton et al. 2010, p. 91), particularly for a government with an austerity agenda. In addition to proposals for ambitious, long-term actions, the review included short term, affordable recommendations facilitating its ability to gain rapid policy traction (notably recommendation 3, to establish 12 Ecological Restoration Zones which was responded to immediately through the *Natural Environment White Paper’s* commitment to establish 12 Nature Improvement Areas).

In addition to illustrating feasibility, the recommendations within the report were inclusive. The review panel included members of conservation NGOs, the National Farmers Union, the Country Land and Business Association and Local Authorities, and at least some of the recommendations in the report resonated with each of these stakeholders. This ensured that there was support for (or at least lack of opposition to) the review from diverse stakeholders, so politicians received no clamour of dissent from influential groups, as is often the case when environmental reports are published. The White Paper noted that a multi-stakeholder approach to landscape-scale conservation was desirable (Table 2).

## Use of clear, accessible language

Interviewees with close knowledge of the *Lawton Review* (e.g. those in DEFRA and Natural England) praised its clear presentation style. For example, a landscape ecologist at Natural England,^6^ argued that the report ‘made a compelling argument, and it was very well presented’. It wisely communicated its findings in an accessible way (Lawton and Rudd 2016). The lead author of the report, Professor Sir John Lawton, commented that:

‘I devoted six weeks of my life to getting it right and writing it in such a way that was understandable, clear and readable by non-expert policy-makers. I wrote a long executive summary to try and make it accessible. I spoke with one of the MPs attached to the environment department after the report. He said he wasn’t going to bother reading the report in full until he saw the word ‘creepy crawly’. He said to me ‘the word creepy crawly^7^ in a scientific report for the government, this can’t be that bad!’ He went on to read it in full.’

Improving the communication of science is commonly recommended to improve the chances of evidence-based policy. In general, policy-makers are not expert in the field of conservation science, and therefore knowledge must be presented carefully. There was strong evidence in the case of the *Lawton Review* that the accessible writing style was a significant factor in maintaining the attention of policy-makers. A civil servant (1) based at DEFRA at the time agreed that the review’s influence was enhanced because ‘it was not purely a scientific bit of work; instead it was written for a much wider audience.’ Furthermore, another DEFRA civil servant (2) argued that it managed to put ‘forward the sorts of ideas that tend to be associated with tree-huggers and sandal-wearers into much more everyday parlance’, and this accessible style was welcomed by policy-makers.

### Rigorous, authoritative science

The *Lawton Review* panel was an authoritative group of experts with diverse knowledge. As well as academics, organisations represented were: Royal Society for the Protection of Birds, The Wildlife Trusts, Natural England, National Farmers Union, a Local Authority, Country Landowners Association, Lake District National Park CEO, and the Planning Inspectorate (as well as an independent member who previously worked for United Utilities). Over the course of the two decades in which landscape-scale conservation was a frequently recommended idea (e.g. Hossell et al. 2000), the quality of scientific evidence improved. For example, Hossell et al. (2000) conducted a similar type of study to the *Lawton Review* a decade previously, but one of Hossell’s co-authors commented:

Lots of things helped Lawton that we weren’t able to say. I think that the large-scale approach to conservation may have been flushed out as a result of advances in knowledge. For example, the MONARCH study clearly illustrated that species would move around and we needed a new, more dynamic approach to conservation globally and in England. (co-author, Hossell et al. 2000)

Indeed, the literature review by Hossell et al. (2000, p. 3) noted that ‘relatively little work had been published that relates directly to the impact of climate change on habitats and species of greatest conservation concern in the UK.’ In the subsequent ten years, quantitative research into the impact of various pressures on nature (e.g. Huntley et al. 2007; Parmesan and Yohe 2003; Walmsley et al. 2007), particularly climate change, improved the knowledge base for landscape-scale conservation. Importantly, there was also growing understanding of the critical role of high quality large core (often protected) areas and ecological networks in conserving wildlife (see Lawton et al. 2010, Sect. 5). Thus, the *Lawton Review* had a richer selection of relevant primary scientific literature on which to base recommendations. This is well illustrated by an analysis of the literature used to support the *Lawton Review* (Lawton et al. 2010). Of 83 references used, just twenty-two were published before 2000 (26.5 %), and therefore Lawton et al. drew predominantly (73.5 %) on work undertaken in the 21st century. Interviewees at all levels of nature governance pointed to the quantitative value of these newer studies, and observable trends, which combined to form an “avalanche of data” (Academic Conservation Scientist A). The improvement in observing climate change impacts was also noted:

New species are arriving in England and this is making conservationists more certain that climate change will have a significant impact and I think we are all more convinced as compared to a decade ago that it is a serious threat as a result of seeing it with our own eyes. (Conservation Officer, The Wildlife Trusts)

Advances in knowledge were also noted by Lawton in interview:

Knowledge was almost entirely from the primary scientific literature. On the committee, we had a range of expertise. It really was from the primary literature and a small number of absolutely key review papers.

In addition to extensive consideration of peer-reviewed literature, novel analyses were conducted for the report, for example in assessments of the performance of England’s site network. The quality of the science in the *Lawton Review* seemed to have some impact on policy based on comments from the Secretary of State for the Environment. In interview for this project, Spelman argued:

We were in receipt of that work [*Lawton Review*] as a new Government and we just thought that the quality of the work was outstanding. It was an example of a very good piece of scientific work on which to base decisions and allocate funds based on a more predictable outcome.

This would imply that the rigour of scientific review, combined with the range and strength of expertise, helped to make the case for landscape-scale conservation more convincing. This rigour has also been an important factor behind the ongoing influence of the review because recent publications (e.g. ADAS 2013; Humphrey et al. 2015) have reinforced the scientific robustness of the review’s conclusions.

### A policy window opens

Kingdon (2003) argued that policy windows regularly open, allowing a particular issue to rise to the top of a government’s agenda. This usually occurs if there is convergence between three ‘process streams’: problems, policies, and politics. In other words, if a pressing problem becomes too serious to ignore, and a policy is proposed that is practical to carry out, combined with a favourable political context, then fertile ground can be created for uptake of new or old ideas. This provides the opportunity for previously ignored knowledge to make an impact. Political events, as well as receptive individuals in power, helped to create a favourable window for the *Lawton Review*.

The Coalition Government assumed Office in May 2010, based on an election campaign in which the Conservatives had promised to become the ‘greenest government ever’, a promise that the *Lawton Review* was able to capitalise upon. The *Lawton Review* was received by government after the ‘forest sell-off’ controversy in which government appeared to be selling public forests to private enterprises, hence restricting public access to nature. Headlines such as ‘Ministers plan huge sell-off of Britain’s forests’ (The Daily Telegraph 2010) dominated newspapers in the Autumn of 2010, with a significant backlash against those politicians who referred to themselves as the ‘greenest ever’. As a DEFRA civil servant (2) argued, the *Natural Environment White Paper* “was still just about at the tail-end of the greenest government ever and there was a sense that something big and visible needed to be done”; thus listening to the advice offered by the *Lawton Review* was certainly ‘big’ and ‘visible’. Another important political driver was the extensive coverage of missed international targets to halt biodiversity loss by 2010 and considerable political activity associated with agreeing new European and international commitments (a new EU biodiversity strategy and new strategic plan under the Convention on Biological Diversity).

All interviewees noted that individuals in power can determine the reception experienced by scientific evidence. The contribution of Caroline Spelman was especially mentioned. Spelman became Secretary of State at DEFRA in May 2010 and was viewed as a good Minister for the environment. Indeed, a civil servant based in DEFRA (2) at the time stated that colleagues were galvanised by “the determination of the Secretary of State that something was going to happen.” Several other interviewees supported this notion, for example a senior member of The Wildlife Trusts’ thought that “Caroline Spelman very quickly got it” post-election because she had a personal interest in the issue. Furthermore, Lawton praised the fact that:

Caroline Spelman cared. She was bothered about what was happening to biodiversity and so was her minister, Richard Benyon. She read the whole *Lawton Review*. I went to see her about 3 weeks after they had got the report and she had clearly read it, it was marked throughout with her handwriting, she had her own copy. (Professor Sir John Lawton)

Therefore, Spelman’s interest in conservation, alongside wider political events, helped to create a favourable policy window for evidence uptake.

## Discussion

Before discussing the implications of this study for conservation scientists, two caveats are worth noting. Firstly, some may question whether the *Lawton Review* was always destined to be impactful because it was directly commissioned by government, and managed to assemble an authoritative group of scientists. In our opinion it would be wrong to argue that this could solely account for the high impact. Many reports similarly commissioned by government never have impact. Randall (2009) argues that the commissioning of an independent review by government is often used as a way of ‘kicking an issue into the long grass’; a way of appearing to take action without actually doing anything. There was a change in government between commission and publication, meaning that Lawton et al. did not report to those who had initially wanted it. Thus, the new government could easily have dismissed the report as belonging to the last administration and distanced themselves from its results.

Secondly, there was an apparent policy window that created fertile ground for evidence uptake. Whilst it certainly improved the chances of uptake, there are instances in which scientific reports have failed to make an impact, despite appearing to have a window of opportunity. Following the RCEP’s influential report on the release of genetically modified organisms to the environment (RCEP 1989), for example, the Commission proposed a system of risk assessment for releases (GENHAZ) based on practices in the chemicals industry (RCEP 1991). The system proved impractical in such a different context, however, and despite a receptive policy environment, the report had little lasting effect (see Owens 2015, p. 142). So it is not enough for a policy window to be open or opening; practical advice must still be ‘do-able’. One could argue that the *Lawton Review* team was aware of an opening window, and therefore placing the emphasis on themes like the economic value of nature, climate change, and localism, was a deliberate way of seizing on the opportunity. The *Lawton Review* framed the long-standing *policy* option of landscape-scale conservation within the increasingly mainstream *problem* of climate change. In presenting a storyline that argued that a new approach to nature conservation would satisfy *political* objectives, the report proactively brought several ‘process streams’ together (Kingdon 2003) *(policies, problems,* and *politics)*, thereby increasing the impact of its scientific recommendations.

In addition, simple communication and better evidence do not fully account for the success of the review. Conservation scientists have sometimes focussed too heavily on making their knowledge understandable. For example, some conservation scientists have dismissed the premise of engaging closely with policy, arguing that the ‘primary duty of scientists to policymakers is to present their work clearly’ (Morecroft et al. 2014, p. 842 in response to Rose 2014a). Whilst this is an important ambition, policy analysts have shown that better communication *per se* rarely leads to evidence uptake (Radaelli 1995; Owens 2015; Lawton 2007). Also, Owens (2015) has illustrated that more certain evidence rarely leads to policy change on its own; rather, knowledge must be persuasive alongside other factors in policy formation (Rose 2014a).

The success of the *Lawton Review* therefore also illustrates the power of presenting a persuasive narrative, alongside clear communication and conducting rigorous science. One might consider it to be a good example of a ‘boundary object’ (Star and Griesemer 1989), since the report helped to bridge the gap between science and policy through clear and relevant communication of a scientifically acceptable idea. Unsurprisingly, therefore, the *Lawton Review* is viewed as a good piece of scientific presentation. Whilst many of its framing strategies were developed to present a convincing scientific argument in an English context, the messages conveyed by its success are relevant much more widely, even to those who are not necessarily directly presenting knowledge to policy-makers. There are three key messages to take forward.

Firstly, there are benefits to framing knowledge in a policy-relevant way. This will not always involve using the storylines of ecosystem services, climate change, and localism, because salient issues change regularly, while political, economic, and social contexts vary by place. However, scientists in all areas can seek to identify the likely future priorities of policy-makers by conducting a horizon scan of the forthcoming legislative plans of government (Sutherland et al. 2015a). When the time comes for each salient issue, scientists should try and be ready to frame their knowledge accordingly. Whilst this might not necessarily fit the logistics of a long-term scientific study, modes of reporting could be developed to account for short, sharp, policy cycles (Hulme et al. 2010).

Secondly, the success of the *Lawton Review* illustrates the usefulness of an inter-disciplinary mind; in other words, one trained in the methods of science *and* the messy realities of the policy-making process (like Lawton). It is useful to suggest inter-disciplinary collaboration as a means of creating evidence-based policy (Young et al. 2014). In the case of the *Lawton Review*, panel representation of a large spectrum of interests helped to secure wide buy-in and support for the recommendations. But collaborations can sometimes be constrained by difficulties in understanding the approaches of different individuals (Pooley et al. 2014) and thus it would be helpful if scientists could draw on inter-disciplinary knowledge to help understand how fellow collaborators work and think.

Finally, it is important to comment on the possible tension created by framing science in a policy-relevant way. Concerns have been raised when scientists partake in policy advocacy, as some consider this to be an erosion of scientific objectivity (Lackey 2007). Instead, researchers may wish to act as a ‘pure scientist’ (Pielke 2007), conducting their research with no involvement in the policy process. There is evidence of conservation scientists who actively partake in so-called defensive boundary work (Gieryn 1983) protecting the hallmarks of science against other activities (such as advocacy) that they would consider as undermining the scientific method (Rose 2014b). However, others argue that a broad spectrum of advocacy exists (Scott et al. 2007). Indeed, Garrard et al. (2015) presents a convincing argument when suggesting that it is a myth that advocacy always harms scientific credibility. Instead, they argue that space should be opened for conservation scientists to engage in debate. Moreover, Pielke (2007) is clear that the role of ‘issue advocate’ should not automatically be viewed with disdain. If scientists are open about their views, then it can be advantageous for them to get involved in the policy-making process (Pielke 2007).

The process of framing science within policy-relevant storylines blurs the line between issue advocacy and honest brokerage of knowledge (Pielke 2007). On the one hand, it means that scientists are trying to present a convincing case for policy change, based on clear scientific knowledge. However, if salient frames are selected after the scientific part of the study has been carried out, the rigour of the scientific method remains intact.^8^ One might say, therefore, that the *Lawton Review* performed the role of an *honest advocate*. Knowledge was first honestly compiled using a rigorous scientific methodology, and by an authoritative selection of experts. The review was then honest in terms of presenting a robust knowledge base, but packaged this in a convincing way to improve the quality of its advocacy. Thus, the *Lawton Review* is justifiably seen as ‘an example of how to present good science to policy-makers’ *(DEFRA civil servant 2)*, and its balance of honest advocacy could be an aim for those interested in improving the chances for evidence-based policy.

## Appendix 1: list of interviewees (35)

(Only Professor Sir John Lawton and Ms Caroline Spelman, MP, are named as it was impossible to anonymise given the extracts used. Permission was gained from the participants for this).

All were present in the role stated at the time of interview unless stated (September 2013–2014).

### Policy-makers or Natural England/Forestry Commission/Environment Agency

The Rt Hon. Caroline Spelman MP, former Secretary of State for Environment, Food and Rural Affairs-12 May 2010—4 September 2012.

- DECC civil servant, Climate scientist.
- DEFRA civil servant (1), Biodiversity Team.
- DEFRA civil servant (2), connected with the Chief Scientific Adviser.
- DEFRA civil servant (3), Local Nature Partnerships.
- DEFRA civil servant (4).
- Environment Agency, Senior Policy adviser.
- Forestry Commission, senior policy adviser.
- Natural England, former climate change specialist (1).
- Natural England, former climate change specialist (2).
- Natural England, landscape-scale conservation expert.
- Natural England, Head of Profession for Biodiversity.
- Natural England, Principal specialist in Landscape Ecology.
- Natural England, Leader of Climate Change Work.

### Local Authority policy-makers

Co-ordinator for Greater Cambridgeshire Local Nature Partnership.

### Scientists involved with the key reports

Professor Sir John Lawton, Chair of *Lawton Review* (Lawton et al. 2010).

Co-author, the Hossell Report (Hossell et al. 2000).

### Conservation scientists

University of Oxford, Senior Research Fellow.

University of Liverpool, Senior conservation biologist.

University of Durham, Senior academic.

British Trust for Ornithology, Principal Ecologist.

University of York, Senior conservation biologist.

### Members of NGO science/policy teams or regional conservation managers

Director, UK conservation NGO.

Butterfly Conservation, Senior Staff member.

RSPB, conservation manager.

RSPB, former Conservation Director.

RSPB, Head of Environmental Research.

RSPB, co-ordinator of Dark Peak Nature Improvement Area.

RSPB, Senior Policy Officer.

The Broads Authority, Head of Strategy and Projects.

The Wildlife Trusts, former Director of the “Living Landscapes” scheme.

The Wildlife Trusts, Regional Chief Executive.

The Wildlife Trusts, Conservation Officer (1).

The Wildlife Trusts, Conservation Officer (2).

Woodland Trust, Senior Policy Adviser.

## Appendix 2 Sample interview questions

Sample interview for a Defra policy-maker—all interviews contained two distinct strands—general science-policy questions, and then a focus on the Lawton Review/White Paper.

- What role do you perform within this policy organisation?
- What is the role of evidence within decision-making about conservation?
- What are the major constraints in using evidence in policy-making?
- How do you approach uncertainty in science in policy discussions?
- Does the source of evidence make a difference?
- Do you think that the Minister in charge of the department makes a difference in evidence use for conservation?
- How important was the issue of climate change within DEFRA?
- How far do you think that climate change became a significant issue for conservation? And when did it become a significant issue?
- To what extent are ecosystem services important for conservation?
- How would you summarise the role of conservation organisations within policy discussions?
- Are some conservation organisations more influential than others?
- Why do you think we had a White Paper on the natural environment in 2011?
- What kind of evidence was influential in the policy decisions laid out in the White Paper?
- Why do you think the *Lawton Review* had such a significant impact on the White Paper?
- Why do you think the *Lawton Review* had a greater impact than previous reports that provided similar recommendations?
- What were the difficulties in presenting an ambitious White Paper in a time of austerity?
- What are the major obstacles to implementing the vision of the White Paper?

## Footnotes

1 As part of this approach, better management of existing protected areas was also recommended.

2 Nature conservation in the UK is a devolved issue and so each constituent part of the UK (Wales, Scotland, England, Northern Ireland) has statutory conservation agencies that are independent from the UK government. These agencies have some powers to make conservation policy referring to their devolved nation, and therefore policies vary between different constituent parts of the UK. The *Natural Environment White Paper* referred only to England.

3 For the first question, the *Lawton Review* was read through three times and each time a different justification for conserving nature was raised, it was noted down. These broadly fit into two themes.

4 For the second question, the *Lawton Review* was again read three times. Each time a different ‘stakeholder’—e.g. National government, local government, NGOs, businesses, farmers—were included as playing a key role in a landscape-scale approach, they were noted down. After compiling a list of all those stakeholders responsible for a landscape-scale approach, it was clear that the *Lawton Review* adopted a local and multi-stakeholder lens to its recommendations.

5 The coding followed the three-stage process suggested by Bryman (2008). Firstly, in initial coding numerous codes were initially attached to the data, sometimes up to a quantity of one code per line. Then, axial coding merged similar codes, before the final stage of selecting the most important codes in the study. The final codes were those mentioned most often in reference to the success of the *Lawton Review*—here, comments about the implementation of the *Lawton Review* were dropped since they were less relevant to the research question.

6 Statutory agency for English nature conservation.

7 ‘Creepy crawly’ is a commonly used UK colloquialism for insects.

8 Of course, it could be argued that the science itself is also susceptible to processes of framing; for example, in the identification of problems and of questions that need to be addressed.
